# Daily Rhythmicity of Muscle-Related and Rhythm Genes Expression in Mackerel Tuna (*Euthynnus affinis*)

**DOI:** 10.3390/biology12091211

**Published:** 2023-09-05

**Authors:** Wenwen Wang, Shiming Dai, Longlong Liu, Zhengyi Fu, Rui Yang, Gang Yu, Zhenhua Ma, Humin Zong

**Affiliations:** 1 Key Laboratory of Efficient Utilization and Processing of Marine Fishery Resources of Hainan Province, Sanya Tropical Fisheries Research Institute, Sanya 572018, China; 2 Tropical Aquaculture Research and Development Center, South China Sea Fisheries Research Institute, Chinese Academy of Fishery Sciences, Sanya 572018, China; 3 Hainan Academy of Ocean and Fisheries Sciences, Haikou 571126, China; 4 College of Science and Engineering, Flinders University, Adelaide, SA 5001, Australia; 5 National Marine Environmental Center, Dalian 116023, China

**Keywords:** mackerel tuna, daily expression, biological clock, related gene expression, muscle

## Abstract

**Simple Summary:**

This study used RT-qPCR to investigate the rhythmicity of muscle rhythmic genes and functional genes associated with mackerel tuna under varying weather conditions. The study revealed that rhythmic and functional genes in mackerel tuna showed daily rhythmicity in their expression levels. These findings indicated that rhythmic genes regulate functional genes in mackerel tuna, which displayed vigorous daily rhythmicity under the influence of weather and time of day. The study will serve as a reference for the artificial breeding of mackerel tuna in the future.

**Abstract:**

The aim of this study was to investigate the circadian rhythm of muscle-related gene expression in mackerel tuna under different weather conditions. The experiment was carried out under two weather conditions at four sampling times (6:00, 12:00, 18:00, and 24:00) to determine the expression of growth, function, and rhythm genes: white muscle rhythm genes were rhythmic on sunny and cloudy days, except for PER3 and RORA; all functional genes had daily rhythmicity. Red muscle had daily rhythmicity on both sunny and cloudy days; functional genes had daily rhythmicity except for MBNL. The expression levels of the rhythm gene PER1 were determined to be significantly different by independent *t*-test samples in white muscle at 6:00, 12:00, 18:00, and 24:00 under different weather conditions; the expression levels of the functional genes MBNL and MSTN were both significantly different. In the red muscle, the expression of the rhythm genes PER3, REVERBA, and BMAL1 was determined by independent *t*-test samples at 6:00, 12:00, 18:00, and 24:00 on cloudy and sunny days; the functional gene MBNL was significantly different. The present study showed that mackerel tuna muscle rhythm genes and functional genes varied significantly in expression levels depending on weather, time of day, and light intensity and that the expression levels of myogenic genes were closely related to clock gene expression. The fish were also able to adapt to changes in light intensity in different weather conditions through positive physiological regulation.

## 1. Introduction

The phenomenon of biorhythms corresponds directly to the cyclical changes in the relative positions of the Earth, sun, and moon. Biological rhythms are perihelion rhythms of circadian rhythms, also known as biological clocks, and biological clock genes regulate the rhythmical nature of an organism’s activity. They are commonly found in the okbiological world in 24 h cycles [1] and are co-regulated, mainly with the external environment, so that the behavioral activities and physiological processes of organisms are coordinated with changes in the external environment [2]. The hypothalamus is the primary controller of the body’s biological rhythms, but peripheral tissues also have circadian rhythms [3], such as muscles. Circadian rhythms are biological clocks based on cell-autonomous transcriptional mechanisms capable of regulating a variety of biological functions within a certain time frame. The generation and maintenance of such rhythms depend on multiple clock genes that work together to maintain the intrinsic rhythms of the organism [4]. Their regulation allows muscles to adapt quickly to changes in their environment by regulating the rhythmic hormones of the endogenous clock [5,6]. Biological clock proteins can act as transcription factors to regulate their gene expression while forming autonomous regulatory loops, including CLOCK, PER, CRY, etc. [7]. It has been shown that circadian rhythms exist in mammals [8,9]. In total, 215 circadian rhythm genes have been identified in mouse skeletal muscle [10]. Currently, the study of circadian rhythms of fish muscles and their effects on physiological activities is in its infancy and has been less studied [11]; it is mainly investigated in pelagic fish, such as zebrafish (*Danio rerio*) and Japanese medaka (*Oryzias latipes*) [12,13].

Muscle is responsible for multiple physiological functions such as movement, metabolism, and endocrinology, which mainly involves the direct regulation of the central biological clock and the self-regulation of its own endogenous internal biological clock [14]. It has been suggested that genes with circadian rhythmicity in mice include not only BMAL1, CRY2, PER2, etc. but also muscle-related functional genes [15]. In muscles, circadian rhythms regulate muscle growth and development, metabolism, and energy production. Muscle development depends mainly on transcriptional activators and repressors. TRIM25 (oncogenic E3 ligase): As an important transcription factor, TRIM25 is involved in the regulation of innate immune responses against viruses [16]. STAT3 is a prominent contributor to cellular immunity and autoantigen tolerance by regulating diverse physiological processes, including cell growth, differentiation, and apoptosis [17]. MBNL is an RNA-binding protein that has important functions in muscle development, regulating the splicing process in both directions, promoting cell proliferation, and inhibiting apoptosis [18,19]. There are two copies of MSTN in muscle tissue, i.e., a single copy and a double copy of the MSTN gene [20]. In vertebrates, at least one MSTN gene exists in a two-copy form [21]. MSTN is a negative regulator of myoblast growth and development [22]. Circadian genes in muscles significantly impact muscle growth and are correlated with the circadian rhythmicity of genes responsible for muscle function. Together, they regulate their muscle fiber growth, metabolism, and biological clock [22].

The mackerel tuna (*Euthynnus affinis*) is a highly migratory offshore pelagic fish, one of the most valuable aquaculture fish on the market, widely distributed in the coastal waters of the Indo-Pacific region [23,24,25]. Despite the economic importance of mackerel tuna, little research has been reported on muscle rhythm genes and functional genes in mackerel tuna. In this regard, we determined the mRNA expression levels of genes responsible for the growth, function, and rhythm of white and red muscles in mackerel tuna. The aim of the study is to elucidate the correlation between the white muscle, red muscle, and rhythm genes of the mackerel tuna, and this analysis will provide good basic data for artificial breeding and disease prevention of mackerel tuna and lay the foundation for subsequent artificial breeding of mackerel tuna.

## 2. Materials and Methods

### 2.1. Animal

The mackerel tuna used in this study were collected from Lingshui Experimental Base, Hainan, China. Mackerel tuna of similar weight (1163 ± 284.6 g) were transferred to an indoor recirculating aquaculture system for more than 6 months of temporary rearing prior to the experiment.

### 2.2. Sampling Procedures

In this study, samples were collected under sunny and cloudy conditions, setting four sampling times (6:00, 12:00, 18:00, 24:00) as a cycle. During the experimental period, the staging conditions of mackerel tuna were air temperature (32.45 ± 2.53) °C, water temperature (32.68 ± 0.67) °C, and dissolved oxygen (7.61 ± 0.07) mg·L^−1^. A cycle was divided into three buckets, and three fish were removed from each of the three buckets at each time point for biological replicates. Light intensity on sunny days was 0.06 μmol/s/m^2^ (6:00), 25.67 μmol/s/m^2^ (12:00), 21 μmol/s/m^2^ (18:00), and 0.02 μmol/s/m^2^ (24:00); on cloudy days it was 0.04 μmoL/s/m^2^ (6:00), 16.05 μmol/s/m^2^ (12:00), 4.42 μmol/s/m^2^ (18:00), and 0.03 μmol/s/m^2^ (24:00). First, the fish were anesthetized using eugenol, and then muscle tissue was rapidly collected. The collected muscle tissues were stored at −80 °C for subsequent RNA extraction.

### 2.3. RNA Extraction and Reverse Transcription

After grinding mackerel tuna muscles, the samples were homogenized by adding 1 mL Trizol (Invitrogen, Carlsbad, CA, USA) per 50–100 mg of tissue and ground thoroughly until no tissue mass was present. Then, 200 μL of chloroform were added to the homogenized sample. The samples were shaken vigorously for 15 s and left at room temperature for 3 min. The samples were centrifuged at 4 °C for 15 min at 12,000× *g*. After centrifugation, the supernatant was added to an equal volume of isopropanol, mixed well, and then centrifuged at 4 °C for 10 min at 12,000× *g* after standing at −20 °C for 20 min. The precipitate at this point was crude RNA; 1 mL of 75% ethanol was added to wash the precipitate at 4 °C with 7500× *g* centrifugation for 5 min. The supernatant in the centrifuge tube was aspirated and air-dried indoors to add an appropriate amount of DEPC water (30–100 μL) to fully dissolve the RNA precipitate. RNA was tested by electrophoresis through an agarose gel. We observed whether the RNA bands extracted in the experiment were clearly visible, and when the ratio of 28S:18S was 2:1 and the A260/A280 was between 1.8 and 2.0 when observed by spectrophotometer, the RNA integrity was considered to be good. The cDNA was obtained by reverse transcription according to the EasyScript^®^ First-Strand cDNA Synthesis SuperMix instructions (TransGen Biotech Co., Ltd., Beijing, China).

### 2.4. Gene Expression Analyses

Related gene sequences were determined based on the mackerel tuna genome gene sequence (non-public data) and design primers using Primer Premier 5 software (version number: 5.0); see Supplementary Material Tables S1 and S2 for full gene names. The GAPDH gene was used as an internal reference. Three replicates of real-time fluorescent quantitative PCR (qRT-PCR) were analyzed for each sample in a 96-well plate using the SYBR Green Kit (Beijing Tangen Biotechnology Co., Ltd., Beijing, China). The 20 μL reaction consisted of 10 μL of 2× RealUniversal PreMix, 0.6 μL of forward and reverse primers (10 μM), 2 μL of cDNA template, and 6.8 μL of RNase-free ddH_2_O. The amplification procedure was 95 °C for 15 min, followed by the cycling of the amplification procedure: denaturation at 95 °C for 10 s, annealing at 56 °C for 20 s, and extension at 72 °C for 30 s in 40 cycles. The amplification efficiency of each gene was 90–110%, and Pearson’s coefficient of determination (R^2^) was >0.97. The relative fluorescence values were calculated according to the 2^−ΔΔCT^ calculated method.

### 2.5. Statistics

Differences between different time points on the same day were analyzed by one-way ANOVA using SPSS software (version number: 18.0.0). Independent sample *t*-tests were used to analyze significant differences between sunny and cloudy days between the same time points, and *p* < 0.05 was considered a significant difference. Two-way ANOVA was performed to test the interaction of different weather conditions and time of day. The presence or absence of rhythmicity was determined using the Acro circadian rhythm analysis program (http://www.circadian.org/softwar.html, accessed on 16 October 2022).

## 3. Results

### 3.1. Analysis of the Rhythmicity of the Muscle Colck Gene in Mackerel Tuna

#### 3.1.1. Rhythmical Analysis of the White Muscle Clock Gene

**Figure 1:**
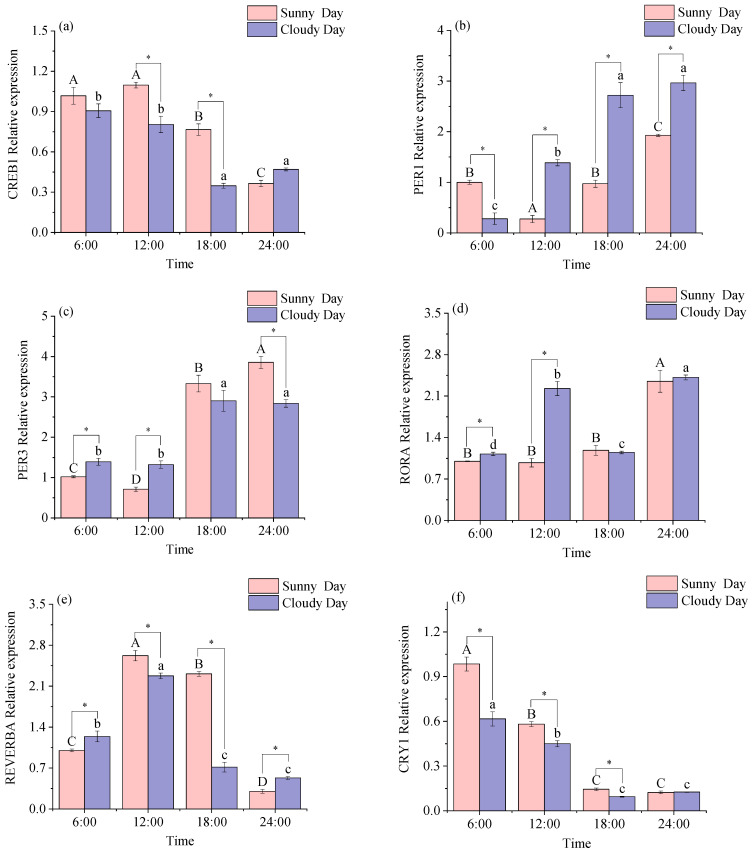

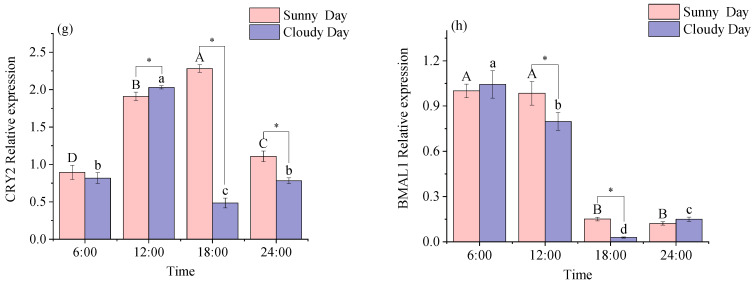
Expression of the white muscle rhythm gene in mackerel tuna at 24 h under different weather conditions. (**a**) *CREB1*; (**b**) *PER1*; (**c**) *PER3*; (**d**) *RORA*; (**e**) *REVERBA*; (**f**) *CRY1*; (**g**) *CRY2*; (**h**) *BMAL1*. The pink color in the figure indicates sunny days, and purple indicates cloudy days. Different letters mean significant differences in ANOVA (*p* < 0.05). Different capital letters mean differences between groups in sunny weather bars (*p* < 0.05), and different lowercase letters mean differences between groups in cloudy weather (*p* < 0.05). * indicates significant differences at the same time in different weather conditions (*p* < 0.05).

As can be seen from Figure 1, all rhythm genes were expressed in white muscle. The expression levels of CREB1, PER1, REVERBA, CRY1, and CRY2 were detected in the white muscle under sunny conditions when the Acro assay was used (*p* < 0.05). Under overcast conditions, the expression levels of CREB1, PER1, REVERBA, CRY1, and BMAL1 had significant daily rhythmicity in white muscle (*p* < 0.05; Table 1).

Figure 1 illustrates the discrepancy in gene expression levels at the same time rhythm. There were significant differences in the expression levels of CREB1 and BMAL1 at 12:00 and 18:00 (*p* < 0.05; a, h). There were significant differences in the expression levels of PER1 and REVERBA at all time points in the comparison (*p* < 0.05; b, e). There were no significant differences in the expression levels of PER3 at 18:00 in the comparison (*p* < 0.05; c). The performance of RORA at 6:00 and 12:00 was significantly different (*p* < 0.05; d). The performance of CRY1 at 24:00 was not significantly different in comparison (f). The performance of CRY2 at 6:00 was not significantly different in the comparison (g).

Two-way ANOVAs are shown in Table S3 in the Supplementary Material. Time main effect was significant (*p* < 0.05); weather main effect was significant for all rhythm genes except PER3 (*p* < 0.05). Time and weather had a significant effect on the expression level of the white muscle rhythm gene (*p* < 0.05).

#### 3.1.2. Rhythmical Analysis of the Red Muscle Clock Gene

**Figure 2:**
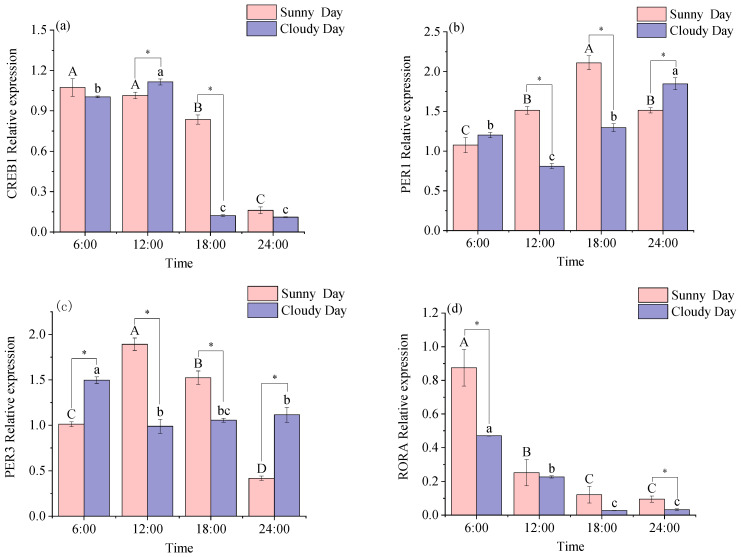

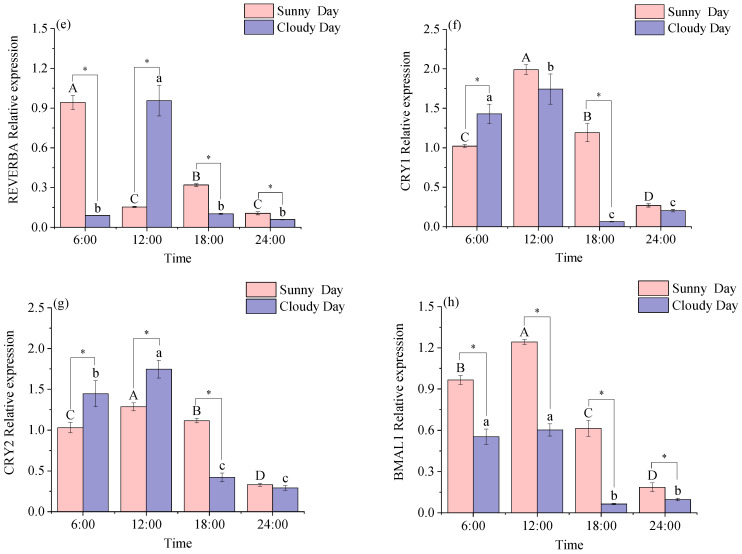
Expression of the red muscle rhythm gene in mackerel tuna at 24 h under different weather conditions. (**a**) *CREB1*; (**b**) *PER1*; (**c**) *PER3*; (**d**) *RORA*; (**e**) *REVERBA*; (**f**) *CRY1*; (**g**) *CRY2*; (**h**) *BMAL1*. The pink color in the figure indicates sunny days, and purple indicates cloudy days. Different letters mean significant differences in ANOVA (*p* < 0.05). Different capital letters mean differences between groups in sunny weather bars (*p* < 0.05), and different lowercase letters mean differences between groups in cloudy weather (*p* < 0.05). * indicates significant differences at the same time in different weather conditions (*p* < 0.05).

Figure 2 shows that all the rhythm genes were expressed in red muscle tissue. However, when analyzing the data with Acro software (version number: 3.5) under sunny conditions, we observed significant daily variations in the expression levels of CREB1, PER1, PER3, RORA, CRY1, CRY2, and BMAL1 in red muscle tissue (*p* < 0.05). In contrast, the expression levels of REVERBA did not show any significant daily rhythmicity in red muscle tissue. Under cloudy conditions, the performance levels of PER1, PER3, RORA, REVERBA, CRY1, and CRY2 had a significant daily rhythm in red muscle (*p* < 0.05); the performance levels of CREB1 and BMAL1 did not have a significant rhythm in red muscle (Table 2).

Figure 2 demonstrates disparities in gene expression related to red muscle rhythms, observed concurrently under distinct weather conditions. CREB1 expression levels were found to be significantly different in the 12:00 and 18:00 comparisons (*p* < 0.05; a). PER1 expression levels at 6:00 were not significantly different in the comparison (*p* < 0.05; b). The expression levels of PER3, REVERBA, and BMAL1 at all times were significantly different in the comparison between the same time points on sunny and cloudy days (*p* < 0.05; c, e, h). The expression levels of ROEA at 6:00 and 24:00 were significantly different between the same time points on sunny and cloudy days (*p* < 0.05; d). The expression levels of CRY1 at 6:00 and 18:00 were significantly different between the same time points on sunny and cloudy days (*p* < 0.05; f). The expression levels of CRY2 at 6:00, 12:00, and 18:00 were significantly different between the same time points on sunny and cloudy days (*p* < 0.05; g).

Two-way ANOVAs are shown in Table S4 in the Supplementary Material. Time main effect was significant (*p* < 0.05); weather main effect was significant for all rhythm genes except CRY2 (*p* < 0.05). Time and weather had a significant effect on red muscle rhythm gene expression levels (*p* < 0.05).

### 3.2. Functional Genes Expression Levels in Mackerel Tuna Muscles

#### 3.2.1. Functional Genes Expression Levels in White Muscle

Table 3 shows that the expression levels of STAT3 and MBNL exhibited a significant daily rhythm in white muscle under sunny conditions (*p* < 0.05). However, the expression levels of TRIM25 and MSTN did not show any significant rhythm in the white muscle. The expression levels of TRIM25 and MSTN were observed to have a significant daily rhythm in white muscle under cloudy conditions (*p* < 0.05). The expression levels of STAT3 and MBNL were not significantly rhythmic in white muscle.

**Figure 3:**
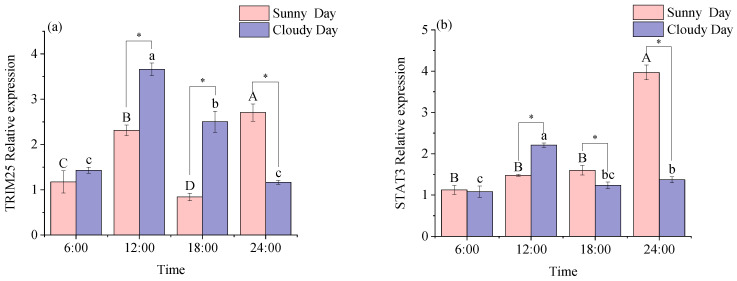

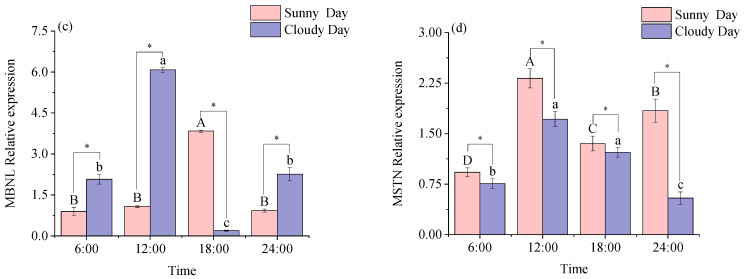
Expression of functional genes in mackerel tuna white muscle under different weather conditions for 24 h. (**a**) *TRIM25*; (**b**) *STAT3*; (**c**) *MBNL*; (**d**) *MSTN*. The pink color in the figure indicates sunny days, and purple indicates cloudy days. Different letters mean significant differences in ANOVA (*p* < 0.05). Different capital letters mean differences between groups in sunny weather bars (*p* < 0.05), and different lowercase letters mean differences between groups in cloudy weather (*p* < 0.05). * indicates significant differences at the same time in different weather conditions (*p* < 0.05).

Figure 3 illustrates the expression levels of functional genes in white muscle at different time points in different weather conditions. The expression levels of the four functional genes in the figure are only not significantly different in the comparison at 6:00 (*p* < 0.05; a, b, c, d).

Two-way ANOVAs are shown in Table S5 in the Supplementary Material. Time and weather had significant effects (*p* < 0.05). Time and weather had a significant effect on white muscle functional gene expression levels (*p* < 0.05).

#### 3.2.2. Functional Genes Expression Levels in Red Muscle

As shown in Table 4, under sunny conditions, STAT3 and MSTN expression levels were significantly daily rhythmic in red muscle (*p* < 0.05); the expression levels of TRIM25 and MBNL were not significantly daily rhythmic in red muscle. Under cloudy conditions, the expression levels of TRIM25, STAT3, MBNL, and MSTN had no significant rhythm in the red muscle.

**Figure 4:**
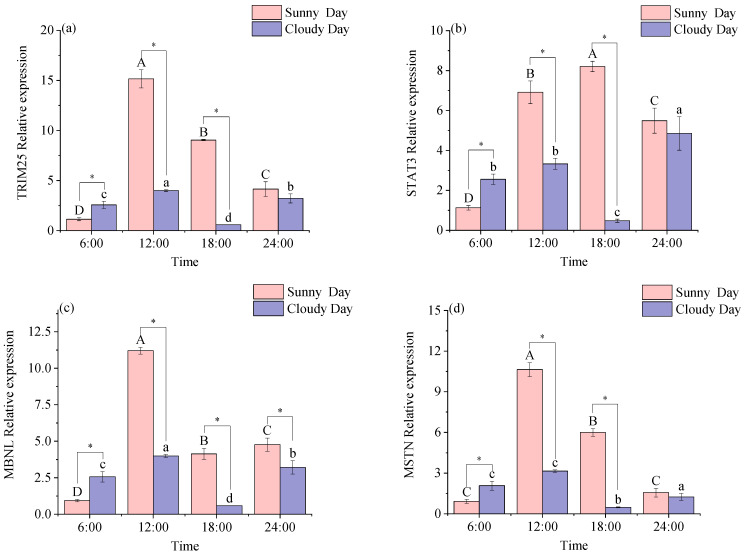
Expression of functional genes in mackerel tuna red muscle at 24 h under different weather conditions. (**a**) *TRIM25*; (**b**) *STAT3*; (**c**) *MBNL*; (**d**) *MSTN*. The pink color in the figure indicates sunny days, and purple indicates cloudy days. Different letters mean significant differences in ANOVA (*p* < 0.05). Different capital letters mean differences between groups in sunny weather bars (*p* < 0.05), and different lowercase letters mean differences between groups in cloudy weather (*p* < 0.05). * indicates significant differences at the same time in different weather conditions (*p* < 0.05).

Figure 4 illustrates the varying changes in red muscle functional genes at the same time point under different weather conditions. There was no significant difference in the expression levels of TRIM25, STAT3, and MSTN at 24:00 between the comparison species (*p* < 0.05; a, b, d). MBNL was significantly different in expression levels at all time points in the same time point comparison (*p* < 0.05; c).

Two-way ANOVAs are shown in Table S6 in the Supplementary Material. Time and weather main effects were significant (*p* < 0.05). Time and weather had a significant effect on red muscle functional gene expression levels (*p* < 0.05).

## 4. Discussion

### 4.1. Muscle Rhythm Gene Expression in Mackerel Tuna

Both circadian rhythms and 24 h periodicity are present in living organisms and are closely related to many physiological functions that are also characterized by circadian oscillations [26]. Vertebrates produce muscle through the regulation of a series of transcription factors that are muscle-specific and control myogenic specification differentiation and repair. CREB1 is an important regulator of muscle differentiation and survival, often acting as a junction and termination point for intranuclear signaling pathways, regulating upstream and downstream signaling molecules, and is closely related to and indispensable for the development of the nervous system [27]. The study found that the expression level of CREB1 in both white muscles had a distinct daily rhythm, and a decrease in expression was observed under both sunny and cloudy conditions. The expression levels of CREB1 in red muscle also showed a decreasing trend, but a clear daily rhythm was only observed under sunny conditions. This indicates that the muscle of skipjack tuna is affected by time, leading to changes in its level of expression, whereas the muscle of red tuna does not exhibit a daily rhythm when it is cloudy due to the weather’s influence, which slows down the phase.

The interaction of PER and CRY proteins in a circadian feedback loop model inhibits transcription activation [5]. It has been shown that only PER2a is daily rhythmic in Atlantic cod, showing no positive correlation with any CRY gene with daily rhythmic expression [28]. In the present study, PER1 and CRY1 had significant daily rhythmicity in muscle, with upregulated expression levels of PER1 and downregulated expression levels of CRY1, with no positive correlation in the genes for circadian rhythmicity. Similar to the study results. It has been shown that the BMAL1 gene activates the PER and CRY genes and that PER and CRY form a complex within the cytoplasm to enter the cell, thereby repressing transcription [29]. The mammalian BMAL shows daily rhythms in the supraoptic nucleus of the optic thalamus [30]. In the present study, BMAL1 in white muscle produced daily rhythmicity under cloudy conditions and BMAL1 in red muscle under sunny conditions, which may be influenced by weather conditions, causing different daily rhythmicity of BMAL1 in red and white muscle of the mackerel tuna under different weather conditions. This suggests that BMAL1 may be an important component of the peripheral clock gene in mackerel tuna muscles. In this experiment, the expression of all the rhythmic genes had small error values at different times. This suggests that all the mackerel tuna were in the same environment when the experiment was conducted. Rhythm genes and the external environment jointly regulate the behavioral activities and physiological processes of the fish to coordinate with the changes in the external environment, which is also a manifestation of the adaptation of the fish to this environment.

### 4.2. Functional Gene Expression in Mackerel Tuna Muscle

Pathogenic infections are fought by the natural immune system using immune molecules and phagocytes. The triple motif family of proteins represents the most extensively researched proteins and possess antiviral properties and aid natural immune regulation. TRIM proteins are a family of proteins that are relatively structurally conserved and have evolved independently and extensively in vertebrates [31]. TRIM25 is a ubiquitin E3-linked enzyme that is involved in signaling, protein processing, promotion of cell proliferation and migration, and the body’s response to antivirals. It has been shown that the TRIM25 protein plays an important role in antiviral processes in vivo in tilapia and grouper [32]. TRIM25 was significantly expressed in rhubarb muscle [33]. In this study, TRIM25 expression levels were highest in both white and red muscle at 12:00 pm under cloudy conditions, which may be due to the fact that light is strongest at this time of the day, making the TRIM25 gene in the mackerel tuna muscle rapidly responsive to immunity.

A member of the STAT protein family, STAT3 primarily functions as a transcription factor and is crucial for the control of immune function, and it can be activated by a number of cytokines and growth factors [34,35,36]. It has been shown that the STAT3 gene is expressed in the muscles of *scleractinian* fish such as zebrafish (Danio rerio) [37], *Siniperca chuatsi* [38], and *Ictalurus punctatus* [39]. In this research, STAT3 expression exhibited a significant daily rhythm in both white and red muscles under sunny conditions and not under cloudy conditions, which could be influenced by lower light intensity experienced in cloudy weather. The condition is capable of regulating the transcriptional regulatory processes of rhythm-related proteins, thereby affecting biological rhythms. Previous studies show that genetic regulation enhances rats’ muscular adaptation to physical activity at night [40]. In the present study, muscle expression levels were relatively high at 12:00 and 18:00 in sunny and cloudy conditions, suggesting that rhythmic genes regulate STAT3 expression during adaptation to light intensity in mackerel tuna, allowing muscle cells to continue to produce energy and thus adapt to daytime exercise.

The MBNL binding protein family plays an important role in muscle and contributes to muscle development. It has been shown that MBNL is expressed in the muscles of Drosophila, mice, and humans [41,42,43]. The marked expression of MBNL in both white and red muscles in this study is in line with the findings. By studying the variation in the daily rhythm display of genes related to muscle growth, a significant daily rhythm was detected in MBNL in the white muscle and MSTN in the red muscle on sunny days. It is likely that rhythmic genes regulate this phenomenon. In Atlantic cod [28], MBNL expression levels were positively correlated with the expression levels of rhythm genes such as BMAL1. In the present study, MBNL expression levels in red muscle under overcast conditions were positively correlated with BMAL1, consistent with the findings that BMAL1 positively regulates MBNL expression in red muscle, possibly acting to maintain muscle cell stability. The experimental error values of MBNL decreased at 12:00 and 18:00 (n = 9), which is essential for fish to consume extra energy to adapt to environmental conditions at this time and to achieve muscle cell stabilization. Therefore, this experiment demonstrates that, at this time, MBNL has a vital role in muscle development among all mackerel tunas that were tested.

Blocking MSTN expression considerably improves muscle development because MSTN is a negative regulator of skeletal muscle growth and reduces muscle cell proliferation and differentiation [44]. It has been shown that CREB1 is regulated by time during its production and plays a key role in muscle growth [45]. In the present study, the CREB1 gene was inversely regulated by MSTN, with an increase in CERB1 expression followed by a decrease in MSTN expression, suggesting that CRBE1 inversely regulates MSTN to regulate muscle growth and differentiation in mackerel tuna.

## 5. Conclusions

In summary, this study investigated the relationship between the daily rhythmic expression of relevant rhythmic genes and functional genes in mackerel tuna and their muscle growth, development, and energy metabolism. All genes exhibited rhythmic expression. The study concluded that light intensity is a crucial environmental factor that regulates and synchronizes the biological clock of mackerel tuna and affects their growth. Light is not the cause of rhythm generation in mackerel tuna, as their growth is maintained by endogenous rhythms. These findings offer a reference point for ongoing research on the influence of circadian genes on muscle physiology.

## Figures and Tables

**Table 1:** Acro and *p*-value analysis of white muscle rhythm genes under different weather conditions.

Genes	Weather	Acro (*p*-Value)	Acrophase
CREB1	S	<0.001	12 ± 0.82
	C	<0.001	6 ± 0.85
PER1	S	<0.001	0 ± 1.00
	C	<0.02	18 ± 1.20
PER3	S	n.s.	n/a
	C	n.s.	n/a
RORA	S	n.s.	n/a
	C	n.s.	n/a
REVERBA	S	<0.005	12 ± 0.98
	C	<0.001	12 ± 0.83
CRY1	S	<0.001	6 ± 0.86
	C	<0.001	6 ± 0.83
CRY2	S	<0.005	18 ± 1.03
	C	n.s.	n/a
BMAL1	S	n.s.	n/a
	C	<0.001	6 ± 0.86

*p* is the statistical difference between statistical discrepancies. Acrophases (circadian peak times) were calculated using a non-linear regression fit based on the cosine function for daily circadian rhythms. The results showed that the phase of circadian rhythms was ±95% confidence interval. n.s., not significant; n/a, not applicable. In the table, “S” denotes a sunny day and “C” denotes a cloudy day.

**Table 2:** Acro and *p*-value analysis analysis of red muscle rhythm genes under different weather conditions.

Genes	Weather	Acro (*p*-Value)	Acrophase
CREB1	S	<0.01	12 ± 1.07
	C	n.s.	n/a
PER1	S	<0.001	18 ± 0.53
	C	<0.001	0 ± 0.56
PER3	S	<0.001	12 ± 0.68
	C	<0.05	6 ± 1.33
RORA	S	<0.005	6 ± 1.04
	C	<0.001	6 ± 0.76
REVERBA	S	n.s.	n/a
	C	<0.02	12 ± 1.17
CRY1	S	<0.001	12 ± 0.31
	C	n.s.	n/a
CRY2	S	<0.001	12 ± 0.69
	C	<0.05	12 ± 1.17
BMAL1	S	<0.001	12 ± 0.66
	C	n.s.	n/a

*p* is the statistical difference between statistical discrepancies. Acrophases (circadian peak times) were calculated using a non-linear regression fit based on the cosine function for daily circadian rhythms. The results showed that the phase of circadian rhythms was ±95% confidence interval. n.s., not significant; n/a, not applicable. In the table, “S” denotes a sunny day and “C” denotes a cloudy day.

**Table 3:** Acro and *p*-value analysis of white muscle functional genes under different weather conditions.

Genes	Weather	Acro (*p*-Value)	Acrophase
TRIM25	S	n.s.	n/a
	C	<0.001	12 ± 0.93
STAT3	S	<0.05	0 ± 1.29
	C	n.s.	n/a
MBNL	S	<0.02	18 ± 1.16
	C	n.s.	n/a
MSTN	S	n.s.	n/a
	C	<0.02	12 ± 1.19

*p* is the statistical difference between statistical discrepancies. Acrophases (circadian peak times) were calculated using a non-linear regression fit based on the cosine function for daily circadian rhythms. The results showed that the phase of circadian rhythms was ±95% confidence interval. n.s., not significant; n/a, not applicable. In the table, “S” denotes a sunny day and “C” denotes a cloudy day.

**Table 4:** Acro and *p*-value analysis of red muscle functional genes under different weather conditions.

Genes	Weather	Acro (*p*-Value)	Acrophase
TRIM25	S	n.s.	n/a
	C	n.s.	n/a
STAT3	S	<0.001	18 ± 0.67
	C	n.s.	n/a
MBNL	S	n.s.	n/a
	C	n.s.	n/a
MSTN	S	<0.02	12 ± 1.2
	C	n.s.	n/a

*p* is the statistical difference between statistical discrepancies. Acrophases (circadian peak times) were calculated using a non-linear regression fit based on the cosine function for daily circadian rhythms. The results showed that the phase of circadian rhythms was ±95% confidence interval. n.s., not significant; n/a, not applicable. In the table, “S” denotes a sunny day and “C” denotes a cloudy day.

## Data Availability

The original contributions presented in the study are included in the article/Supplementary Material. Further inquiries can be directed to the corresponding authors.

## Supplementary Materials

The following supporting information can be downloaded at: https://www.mdpi.com/article/10.3390/biology12091211/s1, Table S1: Gene primers used for qRT-PCR; Table S2: Primer names; Table S3: Effect of light intensity and duration on white muscle rhythm genes in mackerel tuna under different weather conditions; Table S4: Effect of light intensity and duration on red muscle rhythm genes in mackerel tuna under different weather conditions; Table S5: Effect of light intensity and duration under different weather conditions on the functional genes of white muscle in mackerel tuna; Table S6: Effect of light intensity and duration under different weather conditions on the functional genes of red muscle in mackerel tuna.
