# Supplementary material for: Daily Rhythmicity of Muscle-Related and Rhythm Genes Expression in Mackerel Tuna (Euthynnus affinis)
Source: Biology (Basel). 2023 Sep 5;12(9):1211. doi: 10.3390/biology12091211 (PMC10525508; doi:10.3390/biology12091211)
Supplement: Supplementary file 1 [file biology-12-01211-s001.zip › biology-2531950-supplementary.pdf]

# Daily Rhythmicity of Muscle-Related and Rhythm Genes Expression in Mackerel Tuna (*Euthynnus affinis*)

**Wenwen Wang [1,2,†], Shiming Dai [1,2,†], Longlong Liu [4,†], Zhengyi Fu [1,2,3], Rui Yang [1,2], Gang Yu [1,2], Zhenhua Ma [1,2,3,*] and Humin Zong [5,*]**

[1] Key Laboratory of Efficient Utilization and Processing of Marine Fishery Resources of Hainan Province, Sanya Tropical Fisheries Research Institute, Sanya 572018, Hainan, China
[2] Tropical Aquaculture Research and Development Center, South China Sea Fisheries Research Institute, Chinese Academy of Fishery Sciences, Sanya 572018, Hainan, China
[3] College of Science and Engineering, Flinders University, Adelaide, SA 5001, Australia
[4] Hainan Academy of Ocean and Fisheries Sciences, Haikou 571126, Hainan, China
[5] National Marine Environmental Center, Dalian 116023, China
* Correspondence: zhenhua.ma@scsfri.ac.cn (Z.M.); hmzong@nmemc.org.cn (H.Z.)
† These authors contributed equally to this work.

**Table S1.** Gene primers used for qRT-PCR.

| Primer name | Sequence (5'-3') | Product size/bp |
|---|---|---|
| TRIM25 | F: CAACATGACCGCCTGATC | 130 |
| | R: TCTTCCCGTCCAACAAAT | |
| STAT3 | F: ACCGCTCGTCTACCTCTA | 124 |
| | R: TAAGTATGGCTGAATAGTGC | |
| MBNL | F: CAGCATCAAGCCAGTCAAA | 106 |
| | R: CAGCTCCGTTGCTCTTCTC | |
| MSTN | F: ACCAGCCCAGAAGACACG | 106 |
| | R: TTCGCAGTTTGCTCAGGATT | |
| CREB1 | F: TGCCCACTCCCATCTATC | 93 |
| | R: CTCCATCTGTGCCGTTATT | |
| PER1 | F: CCAAAGGCGGTTCAGTTA | 144 |
| | R: GAGGCTTCTTGTCTCCCAC | |
| PER3 | F: TCATCGGACGGCATAAAG | 85 |
| | R: TGGGTGACTGGGAAATACTC | |
| RORA | F: CTGGATAGGGTGGGTGGAA | 84 |
| | R: CGTTGGCCCGGATTAGAG | |
| REVERBA | F: CCTACAACCATCCCACAG | 88 |

| | | |
|---|---|---|
| | R: ACCTTACATAGAAGCACCATA | |
| CRY1 | F: GTGGGCAGCCTCCTCTTA | 145 |
| | R: CCGTACTTGTCTCCGTGGTC | |
| CRY2 | F: CTACATGAAGCTCCGTAAGC | 108 |
| | R: CGGTCAAAGTTTGGGTTG | |
| BMAL1 | F: CGTCCAGTGGTAATGTCA | 176 |
| | R: CATGAGTGCTTCTCCTCC | |
| GAPDH | F: ACACTCACTCCTCCATCTTTG | 100 |
| | R: TTGCTGTAGCCGAACTCAT | |

**Table S2.** Primer names.

| Genes | Full names of target genes |
|---|---|
| TRIM25 | Tripartite motif containing 25 |
| STAT3 | Signal transducer and activator of transcription 3 |
| MBNL | Muscleblind like |
| MSTN | Myostatin |
| CREB1 | cAMP responsive element binding protein 1 |
| PER1 | Period 1 |
| PER3 | Period 3 |
| RORA | RAR-related orphan receptor A |
| REVERBA | Nuclear receptor subfamily 1 group D member 1 |
| CRY1 | Cryptochrome 1 |
| CRY2 | Cryptochrome 2 |
| BMAL1 | Brain and muscle arnt-like 1 |
| GAPDH | Glyceraldehyde-3-phosphate dehydrogenase |

**Table S3.** Effect of light intensity and duration on white muscle rhythm genes in mackerel tuna under different weather conditions.

| Genes | ***P* value** | | |
|---|---|---|---|
| | **Time** | **Weather** | **Interactions** |
| CREB1 | < 0.001 | < 0.001 | < 0.001 |
| PER1 | < 0.001 | < 0.001 | < 0.001 |
| PER3 | < 0.001 | 0.067 | < 0.001 |
| RORA | < 0.001 | < 0.001 | < 0.001 |
| REVERBA | < 0.001 | < 0.001 | < 0.001 |
| CRY1 | < 0.001 | < 0.001 | < 0.001 |
| CRY2 | < 0.001 | < 0.001 | < 0.001 |
| BMAL1 | < 0.001 | 0.011 | 0.002 |

Results of two-way ANOVA with SPSS for measuring factors. When interactions are significant ($P < 0.001$), between-group comparisons and independent samples t-tests at the same time point will be used. The same as below.

**Table S4.** Effect of light intensity and duration on red muscle rhythm genes in mackerel tuna under different weather conditions.

| Genes | ***P* value** | | |
|---|---|---|---|
| | **Time** | **Weather** | **Interactions** |
| CREB1 | < 0.001 | < 0.001 | < 0.001 |
| PER1 | < 0.001 | < 0.001 | < 0.001 |
| PER3 | < 0.001 | 0.038 | < 0.001 |
| RORA | < 0.001 | < 0.001 | < 0.001 |
| REVERBA | < 0.001 | 0.001 | < 0.001 |
| CRY1 | < 0.001 | < 0.001 | < 0.001 |
| CRY2 | < 0.001 | 0.269 | < 0.001 |
| BMAL1 | < 0.001 | 0.001 | < 0.001 |

**Table S5.** Effect of light intensity and duration under different weather conditions on the functional genes of white muscle in mackerel tuna.

| Genes | ***P* value** | | |
|---|---|---|---|
| | **Time** | **Weather** | **Interactions** |
| TRIM25 | < 0.001 | < 0.001 | < 0.001 |
| STAT3 | < 0.001 | < 0.001 | < 0.001 |
| MBNL | < 0.001 | < 0.001 | < 0.001 |
| MSTN | < 0.001 | < 0.001 | < 0.001 |

**Table S6.** Effect of light intensity and duration under different weather conditions on the functional genes of red muscle in mackerel tuna.

| Genes | ***P* value** | | |
|---|---|---|---|
| | **Time** | **Weather** | **Interactions** |
| TRIM25 | < 0.001 | < 0.001 | < 0.001 |
| STAT3 | < 0.001 | < 0.001 | < 0.001 |
| MBNL | < 0.001 | < 0.001 | < 0.001 |
| MSTN | < 0.001 | < 0.001 | < 0.001 |
